# Liraglutide, 7,8-DHF and their co-treatment prevents loss of vision and cognitive decline in a Wolfram syndrome rat model

**DOI:** 10.1038/s41598-021-81768-6

**Published:** 2021-01-26

**Authors:** Kadri Seppa, Toomas Jagomäe, Kaia Grete Kukker, Riin Reimets, Marko Pastak, Eero Vasar, Anton Terasmaa, Mario Plaas

**Affiliations:** 1 Institute of Biomedicine and Translational Medicine, Laboratory Animal Centre, University of Tartu, 14B Ravila Street, 50411 Tartu, Estonia; 2 Eye Clinic of Tartu University Hospital, L Puusepa 8, 50406 Tartu, Estonia; 3 Institute of Biomedicine and Translational Medicine, Department of Physiology, University of Tartu, 19 Ravila Street, 50411 Tartu, Estonia

**Keywords:** Diseases of the nervous system, Learning and memory, Molecular neuroscience, Neural ageing, Regeneration and repair in the nervous system, Metabolic disorders, Biomarkers, Optic nerve diseases, Neurological disorders, Neurodegeneration, Neurodegenerative diseases

## Abstract

Wolfram syndrome (WS) is a monogenic progressive neurodegenerative disease and is characterized by various neurological symptoms, such as optic nerve atrophy, loss of vision, cognitive decline, memory impairment, and learning difficulties. GLP1 receptor agonist liraglutide and BDNF mimetic 7,8-dihydroxyflavone (7,8-DHF) have had protective effect to visual pathway and to learning and memory in different rat models of neurodegenerative disorders. Although synergistic co-treatment effect has not been reported before and therefore the aim of the current study was to investigate liraglutide, 7,8-DHF and most importantly for the first time their co-treatment effect on degenerative processes in WS rat model. We took 9 months old WS rats and their wild-type (WT) control animals and treated them daily with liraglutide, 7,8-DHF or with the combination of liraglutide and 7,8-DHF up to the age of 12.5 months (n = 47, 5–8 per group). We found that liraglutide, 7,8-DHF and their co-treatment all prevented lateral ventricle enlargement, improved learning in Morris Water maze, reduced neuronal inflammation, delayed the progression of optic nerve atrophy, had remyelinating effect on optic nerve and thereby improved visual acuity in WS rats compared to WT controls. Thus, the use of the liraglutide, 7,8-DHF and their co-treatment could potentially be used as a therapeutic intervention to induce neuroprotection or even neuronal regeneration.

## Introduction

Wolfram syndrome (WS) is a rare monogenic neurodegenerative disease and is mainly characterized by diabetes mellitus, optic nerve atrophy, and neurodegeneration^1,2^. All the previously mentioned symptoms are also present in the WS rat model as described by our research group^3^. We have shown that GLP1 receptor agonist liraglutide has a beneficial effect in the WS rat. Early treatment with liraglutide was effective to prevent the development of diabetic phenotype in WS rats^4^. Furthermore, 6-month liraglutide treatment reduced neuroinflammation, cellular stress and excitotoxicity in the brainstem of the aged WS rats^5^. So far, all conducted animal experiments have shown that liraglutide delays WS progression but unfortunately, liraglutide does not restore already lost functions. Therefore, additional supporting therapies to liraglutide that are capable to reverse the course of disease are essential to develop.

Recent research has shown that neurotrophic factors are potential molecules that could inhibit or even reverse neurodegeneration. Brain-derived neurotrophic factor (BDNF) activates tropomyosin related kinase B (TrkB) membrane receptors and thereby is involved in the regulation of neuronal development, synaptic plasticity, and protection from oxidative stress and apoptosis^6^. However, BDNF use is limited in clinical practice because its short in vivo half-life and its inability to cross the blood–brain barrier^7^. Hence, small molecules acting as BDNF mimetics have been characterized. 7,8-dihydroxyflavone (7,8-DHF) was found as a naturally occurring flavone acting as a TrkB receptor agonist^8^. In contrast to earlier findings 7,8-DHF was recently found to be unable to reproduce TrkB receptor activation and TrkB-dependent downstream signaling in vitro. Therefore, it was suggested that in vivo 7,8-DHF induced transactivation of TrkB is possibly mediated through other pathways^9^. Regardless of the molecular pathway, since the discovery of 7,8-DHF several studies have confirmed 7,8-DHF neuroprotective effect in vitro and in vivo. In retinal cells 7,8-DHF treatment protected against excitotoxicity and oxidative stress^10^, ameliorated high-glucose induced diabetic apoptosis^11^ and protected immature retinas against hypoxic-ischemic injury via Müller glia regeneration and MAPK/ERK activation^12^. Additionally, it has been shown that in vivo 7,8-DHF improves the spatial learning and memory in cognitively impaired aged rats^13^ and prevents synaptic loss and memory deficits in a mouse model of Alzheimer’s disease^14^. The therapeutic value of neurotrophic factors has never been reported in connection with WS and therefore the aim of this study was to investigate potential therapeutic effect of 7,8-DHF alone, or in combination with liraglutide in a rat model of WS.

## Results

### 3.5-month treatment with the GLP-1 receptor agonist liraglutide delayed the progression of hyperglycemia in Wfs1 KO animals

**Figure 1:**
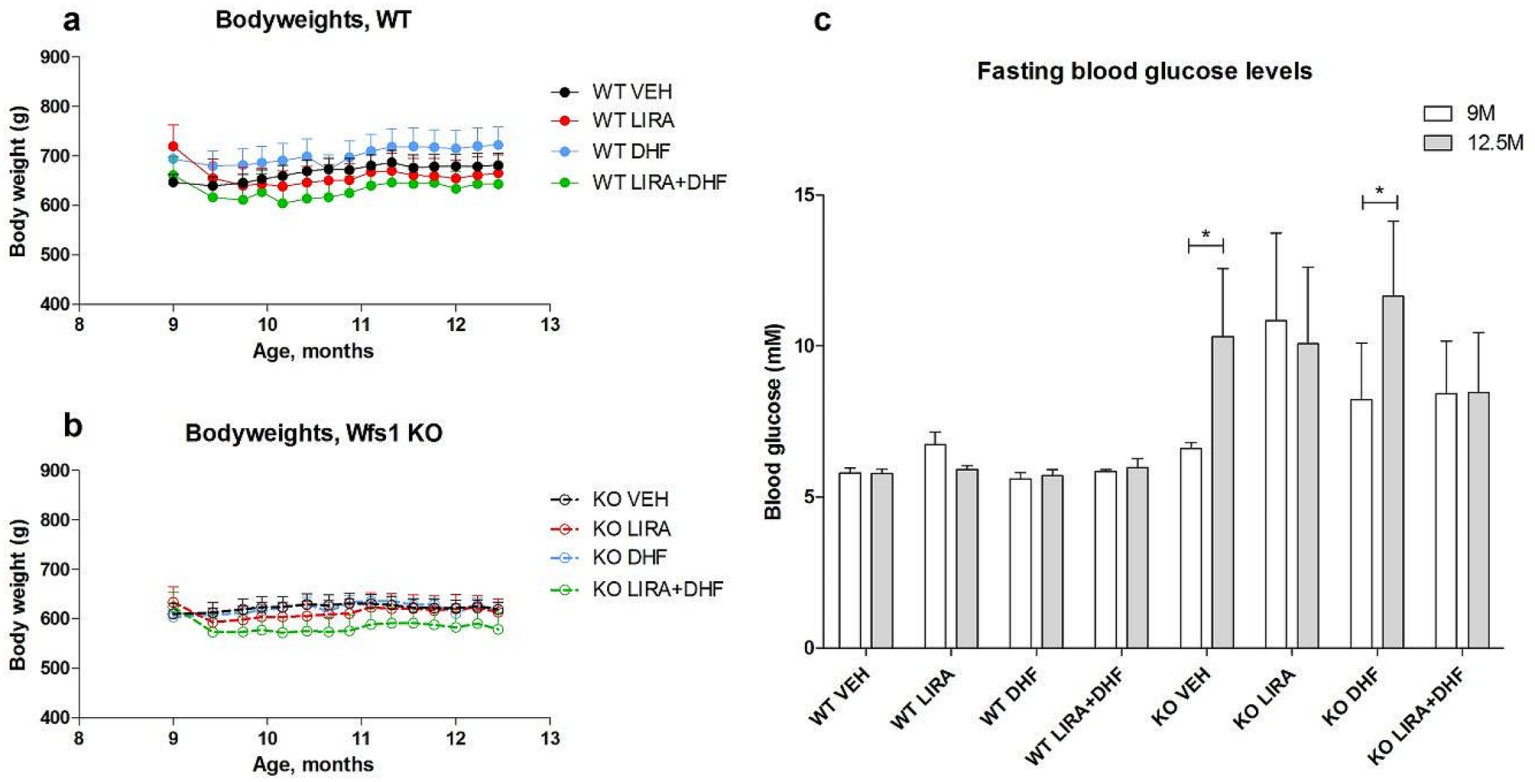
Body weight change and fasting blood glucose levels during the experiment. Liraglutide (LIRA) and liraglutide in combination with 7,8-DHF (DHF) induced a slight decrease of bodyweight at the beginning of experiment in both genotypes (**a**, **b**). Animals were fasted for 3 h before measurement of blood glucose levels. Fasting blood glucose levels were not changed during the experiment in WT animals regardless of the treatment (**c**). As expected, blood glucose levels were elevated in Wfs1 KO animals receiving vehicle treatment (**c**), treatment with 7,8-DHF did not prevent such an increase. In contrast, treatment with liraglutide alone or in combination with 7,8-DHF prevented development of fasting hyperglycemia in Wfs1 KO animals (**c**). The data were compared using repeated measures ANOVA followed by Bonferroni post hoc tests; *p < 0.05. The data are presented as the mean ± SEM, n = 5–8 per group.

In agreement with previous studies^4,5^ liraglutide treatment induced a slight decrease of body weight during the first week of treatment in animals of both genotypes (Fig. 1a,b), this effect of liraglutide was not affected by 7,8-DHF co-treatment. Treatment with 7,8-DHF alone had no effect on body weight (Fig. 1a,b).

As expected, and in agreement with previous studies^3–5^, older Wfs1 KO rats developed fasting hyperglycemia (p < 0.05) (Fig. 1c) and treatment with 7,8-DHF was not preventing an increase in blood glucose levels. However, treatment with liraglutide or with liraglutide in combination with 7,8-DHF prevented the development of hyperglycemia in Wfs1 KO rats (Fig. 1c).

### Co-treatment with liraglutide and 7,8-DHF improved visual acuity in Wfs1 KO rats

**Figure 2:**
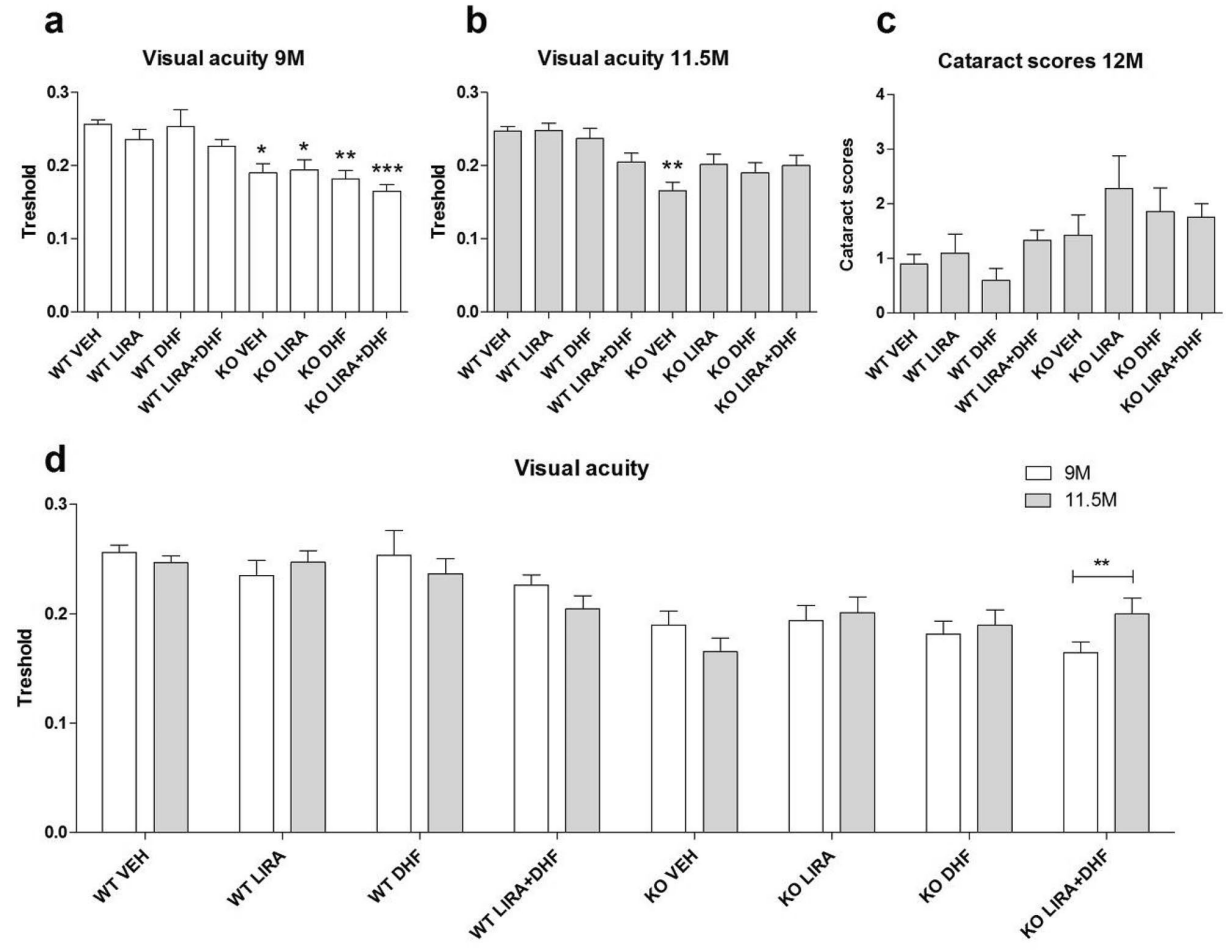
Visual acuity in liraglutide (LIRA), 7,8-DHF (DHF) and liraglutide in combination with 7,8-DHF (LIRA + DHF) treated WT and Wfs1 KO rats. (**a**) Optokinetic reflex response at the age of 9 months, (**b**) 11.5 months. Optokinetic response was measured for both eyes and data is averaged. (**a**) At the age of 9 months, Wfs1 KO VEH animals’ visual acuity was lower compared to WT VEH animals. (**b**) After 2.5 months Wfs1 VEH animals condition worsened, while Wfs1 KO animals treated with liraglutide or 7,8-DHF stayed at the same level. (**c**) Cataract scores at the age of 11.5 months. (**d**) Visual acuity repeated measures, liraglutide in combination with 7,8-DHF (LIRA + DHF) improved Wfs1 KO animals’ visual acuity. The data were compared using one-way or repeated measures ANOVA followed by Bonferroni post hoc tests; *p < 0.05; **p < 0.01; ***p < 0.001 compared to WT vehicle treated animals. The data are presented as the mean ± SEM, n = 5–8 per group.

Visual acuity as measured by optokinetic response was lower in 9 months old Wfs1 KO rats as compared to WT rats of the same age (p < 0.05) (Fig. 2a). At the age of 11.5 months the difference between genotypes increased (p < 0.01) (Fig. 2b). Treatment with liraglutide, 7,8-DHF or with their combination kept Wfs1 KO animals’ visual acuity at the same level with WT control animals (Fig. 2b). There were no statistically significant changes in the cataract score (Fig. 2c). Surprisingly, repeated measures ANOVA revealed that co-treatment with liraglutide and 7,8-DHF improved visual acuity in Wfs1 KO rats during the treatment (p < 0.01) (Fig. 2d). Following Morris water maze experiment was performed under the assumption that no animals used in this study had lost their vision.

### 3.5-month treatment with liraglutide, 7,8-DHF or with their combination delayed the progression of optic nerve atrophy in Wfs1 KO animals

**Figure 3:**
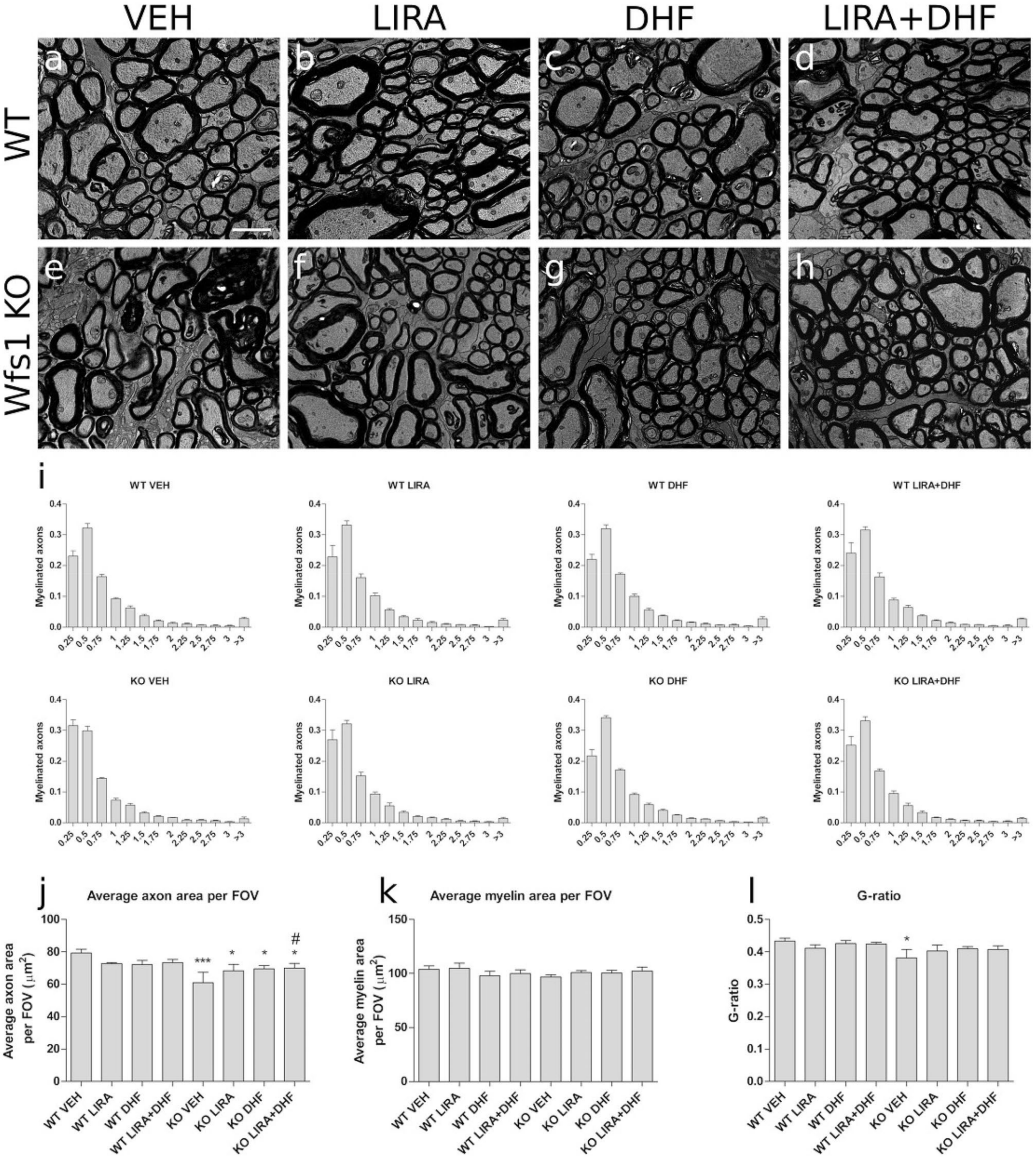
3.5-month treatment with liraglutide, 7,8-DHF or with their combination delayed the progression of optic nerve atrophy in Wfs1 KO animals. Electron micrographs representing cross-sections from analyzed (**a**–**d**) WT and (**e**–**h**) Wfs1 KO rat’s optic nerves from corresponding treatments, liraglutide (LIRA), 7,8-DHF (DHF) or liraglutide and 7,8-DHF (LIRA + DHF). (**i**) Optic nerve axon size distribution in every treatment group, (**j**) average axon area per field of view (FOV), (**k**) average myelin area per FOV, (**l**) G-ratios calculated as total axonal area divided by total axonal area plus myelin area. Quantitative data were compared using factorial ANOVA followed by Fisher’s LSD tests; *p < 0.05; ***p < 0.001 compared to WT vehicle animals and # p < 0.05 compared to KO Sal animals. The data are presented as the mean ± SEM, n = 4–7 per group. Scale bar: 2 μm.

To reveal ultrastructural changes in optic nerves, transmission electron microscopy (TEM) was performed, and illustrative micrographs are seen in Fig. 3. To evaluate changes in optic nerve axons’ size we performed optic nerve axon size distribution analysis (Fig. 3i). Vehicle treated Wfs1 KO animals have significant increase of smaller axons with average axon size 0.25 μm^2^ and decrease of larger axons with average axon size > 3 μm^2^. Treatment with liraglutide, 7,8-DHF or liraglutide and 7,8-DHF sifted the size distribution to WT animals levels, whereas axon calibers larger than 3 μm^2^ could not be rescued.

Optic nerve fiber consists of axon and of the surrounding myelin sheath. To evaluate area-based g-ratio, both the axon area and the surrounding myelin area were measured. Optic nerve axons area per field of view (FOV) was most drastically decreased in vehicle treated Wfs1 KO rats (p < 0.001) (Fig. 3j). Treatment with liraglutide, 7,8-DHF or their combination prevented such decrease (p < 0.05). Moreover, Wfs1 KO rats treated with the combination of liraglutide and 7,8-DHF showed increased axon area per FOV compared to vehicle treated Wfs1 KO animals (p < 0.05) (Fig. 3j). Myelin area per FOV was not changed between groups (Fig. 3k). Area based g-ratio (axon area divided by axon area plus myelin area) was lower in vehicle treated Wfs1 KO rats compared to vehicle treated WT animals (p < 0.05) (Fig. 3l). As myelin area was not changed, G-ratio decrease could be the result of axonal degeneration that is also confirmed by reduced axon area per FOV.

### 3.5-month treatment with liraglutide, 7,8-DHF or liraglutide and 7,8-DHF prevents optic nerve fiber degeneration and induces remyelination

**Figure 4:**
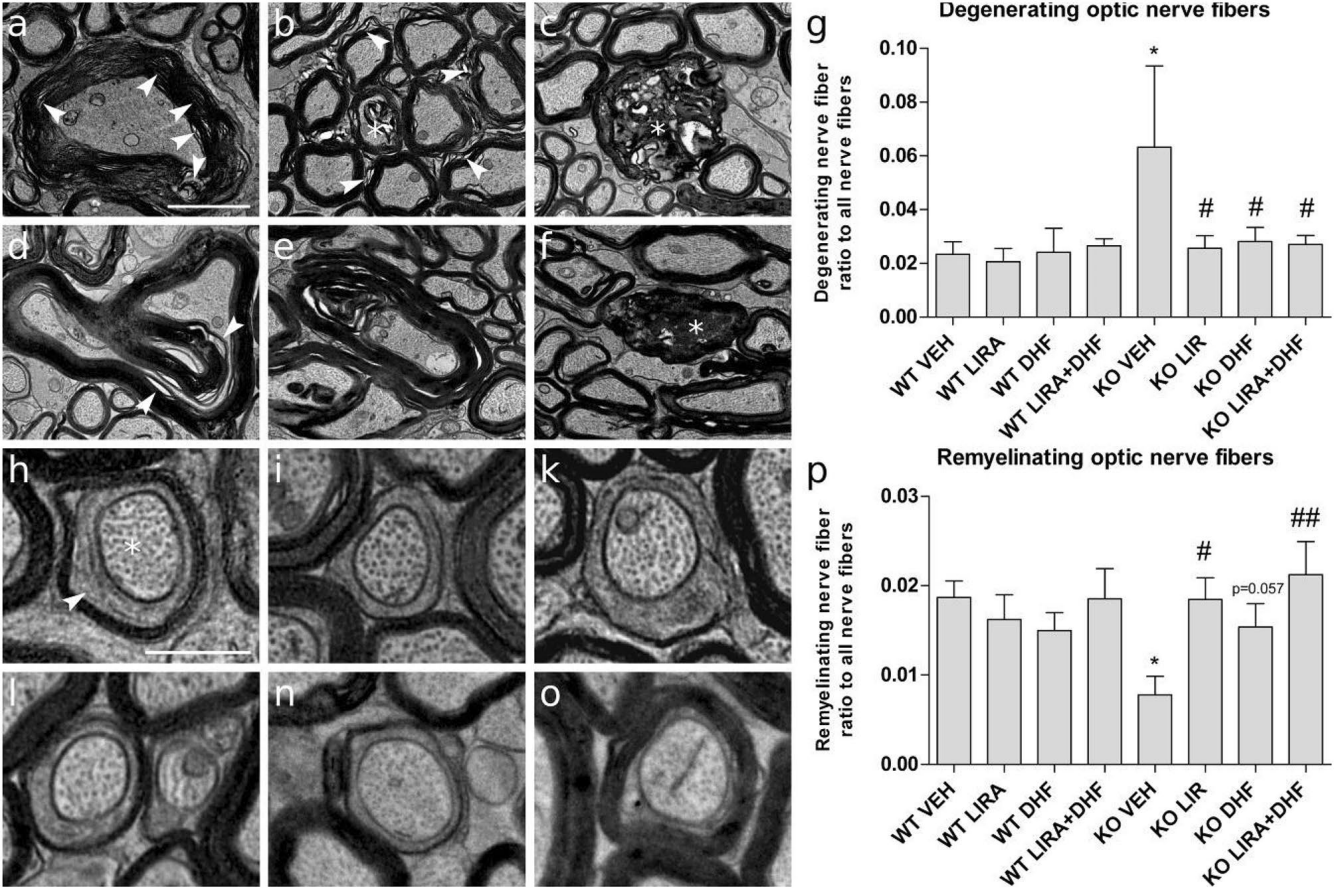
3.5-month treatment with liraglutide, 7,8-DHF or liraglutide and 7,8-DHF prevents optic nerve fiber degeneration and induces remyelination. (**a**–**f**) Wfs1 KO VEH axons with several pathological features. (**a**, **b**, **d**) intense myelin damage is observable as lamina decompaction in small and large caliber axons (arrowheads). (**d**, **e**) axon compression accompanies myelin lamina decompaction. (**b**, **c**, **f**) nerve fibers show severe myelin disruption coinciding with features of axonal degeneration (asterisks). (**g**) Ratio of degenerating fibers to all counted fibers from corresponding treatments. (**h**–**o**) Images illustrate analyzed remyelinating axons (asterisk) where compacted myelin sheath is observable (arrowhead). (**p**) Ratio of remyelinating optic nerve fibers to all counted fibers from corresponding treatments. Quantitative data were compared using factorial ANOVA followed by Fisher’s LSD tests; *p < 0.05 compared to WT vehicle animals and # p < 0.05; ## p < 0.01 compared to KO Sal animals. The data are presented as the mean ± SEM, n = 4–7 per group. Scale bars: (**a**–**f**) 2 μm; (**h**–**o**) 1 μm.

Our next goal was to determine degenerating fibers with severe pathological phenotypes based on criteria described in methods sections (Fig. 4a–f). We observed no difference between WT animals treatment groups (Fig. 4g). The ratio of optic nerve fiber with a pathological alteration in the myelin and axonal structure is significantly increased in Wfs1 KO vehicle group compared to WT vehicle (p < 0.05). Treatment with liraglutide, 7,8-DHF or liraglutide and 7,8-DHF reduced the ratio of degenerating optic nerve fibers compared to vehicle treated Wfs1 KO animals (p < 0.05) which indicates a protective effect of all treatments (Fig. 4g).

In order to quantify remyelinating axons, the optic nerve fibers must display the following distinctive features: few myelin lamellae and the large inner tongue process containing decompacted myelin^15^ as presented on Fig. 4 micrographs h–o. In WT animals, the ratio of remyelinating fibers stayed relatively constant between different treatments (Fig. 4p). Vehicle treated Wfs1 KO rats optic nerves display a significantly lower ratio of remyelinating axons compared to WT animals (p < 0.05) (Fig. 4p). Liraglutide treatment increased remyelinating optic nerve fibers in Wfs1 KO animals when compared to vehicle treatment (p < 0.05) (Fig. 4p). 7.8-DHF treatment also tend to increase remyelinating optic nerve fibers in Wfs1 KO animals (p = 0.057) (Fig. 4p). The co-treatment with liraglutide and 7,8-DHF increased remyelinating optic nerve fibers at most in Wfs1 KO animals (p < 0.01) (Fig. 4p).

### Wfs1 KO rats show impairment in learning

**Figure 5:**
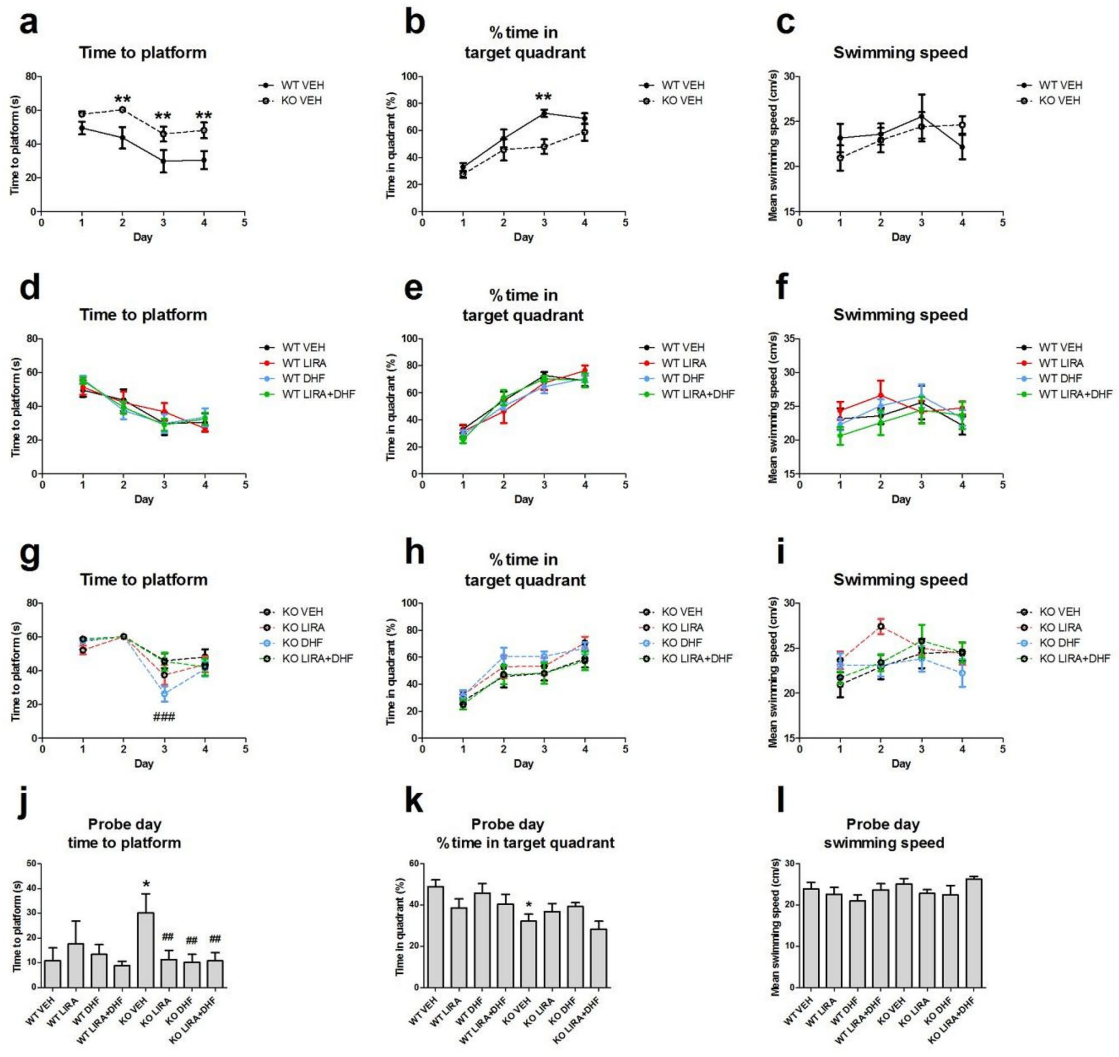
Evaluation of spatial learning of Wfs1 KO and WT rats using Morris water maze. Wfs1 KO animals took longer time than WT littermates to learn the position of the hidden platform (**a**). Similarly, Wfs1 KO rats spent less time in target quadrant than WT rats during training phase (**b**). Swimming speed was not different between genotypes (**c**). Pharmacological treatment with liraglutide (LIRA), 7,8-dihydroflavone (DHF) or their combination (LIRA + DHF) had no overall effect on performance of rats during learning phase (**d**–**i**). On probe day, time to reach the platform was increased for Wfs1 KO rats as compared to WT rats, treatment with liraglutide, 7,8-DHF or their combination improved performance during probe day (**j**). Time spent in target quadrant (**k**) during probe day was decreased in Wfs1 KO animals and swimming speed (**l**) during probe day was not affected by genotype or treatment. Data are presented as mean ± SEM, n = 5–8. Data are analyzed with repeated measures ANOVA followed by Fisher LSD post hoc test, *p < 0.05 and **p < 0.01 compared to WT vehicle treated animals, ##p < 0.01 , ### p < 0.001 compared to vehicle treated Wfs1 KO animals.

In Morris water maze experiment, during training session Wfs1 KO rats needed more time to reach the platform as compared to WT rats (p < 0.01) (Fig. 5a). In WT animals drug treatment had no effect on the performance (Fig. 5d). Time to reach the platform was decreased in 7,8-DHF treated Wfs1 KO animals compared to vehicle treated Wfs1 KO animals during training session (p < 0.01, Fig. 5g). In agreement with an increased time to reach the platform during training session, Wfs1 KO rats spent also less time in the target quadrant as compared to WT rats (p < 0.01) (Fig. 5b). Drug treatments had no effect on the percent of time spent in the target quadrant during training phase in rats of both genotypes (Fig. 5e,h). There were no statistically significant changes in the swimming speed during the training session of Morris water maze experiment regardless of genotype and treatment (Fig. 5c,f,i). On the probe day of Morris water maze, Wfs1 KO rats from the vehicle group spent more time searching for the platform as compared to WT vehicle treated rats (p < 0.05) (Fig. 5j). Liraglutide, 7,8-DHF or their combination improved Wfs1 KO animals’ performance (p < 0.01) (Fig. 5j). Wfs1 KO rats spent significantly less time in target quadrant as compared to WT animals (p < 0.01) (Fig. 5k). In agreement with the training session, on the probe day there were no changes in swimming speed regardless of the genotype (Fig. 5l). In summary, the results from Morris water maze suggest that Wfs1 KO rats indicate impairment in learning as shown by an increase in the time spent searching the platform (Fig. 5a,j) and a decrease in percentage of time spent in the target quadrant (Fig. 5b,k). Importantly, liraglutide, 7,8-DHF or their combination improved Wfs1 KO animals’ performance during the probe day.

### Wfs1 KO rats show lateral ventricles enlargement

**Figure 6:**
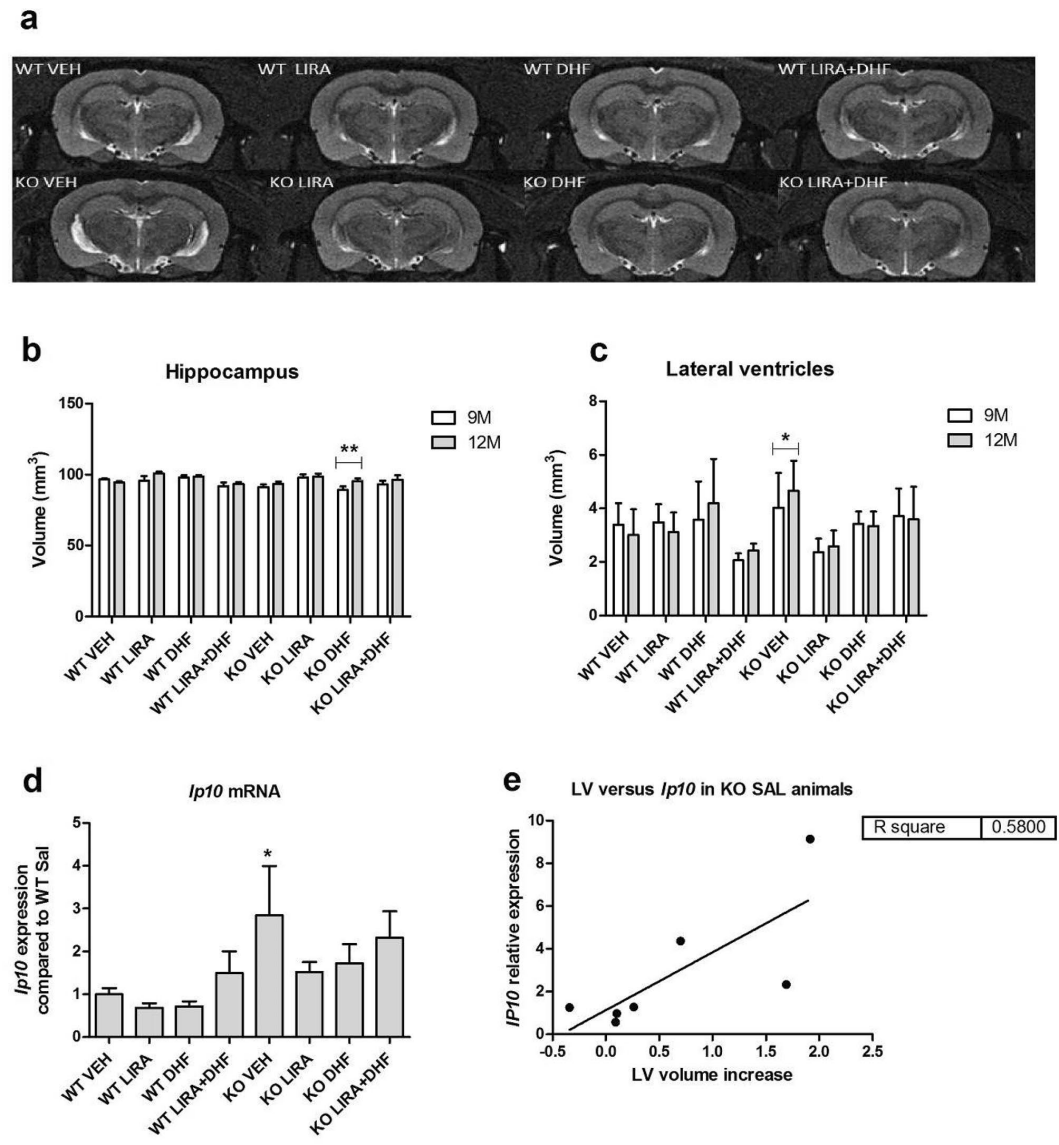
Wfs1 KO rats show lateral ventricles enlargement (**a**) Representative T2-weighted MR images of the hippocampus and lateral ventricles of WT and Wfs1 KO animals treated with liraglutide (LIRA), 7,8-DHF (DHF) or liraglutide and 7,8-DHF (LIRA + DHF). Quantitative MRI analysis of (**b**) hippocampus and (**c**) lateral ventricles. Gene expression analyses of hippocampus were performed to measure (**d**) *Ip10* mRNA levels. (**e**) Correlation between lateral ventricles volume and *Ip10* mRNA expression. Wfs1 KO rats show lateral ventricles enlargement (**a**, **c**), elevated inflammatory marker *Ip10* levels (**d**) and moderate correlation between inflammatory marker I*p10* and lateral ventricles enlargement (**e**). Treatment with liraglutide, 7,8-DHF or with liraglutide and 7,8-DHF combination prevented from ventricular enlargement and hippocampus from cellular stress. Volumes were manually traced by an observer blinded to the genotypes of the rats from T2-weighted MR images using ITK-SNAP software. The MRI data were compared using repeated measures ANOVA followed by Bonferroni post hoc tests; *p < 0.05; **p < 0.01 and gene expression data were compared using factorial ANOVA followed by Fisher’s LSD tests; *p < 0.05. The data are presented as the mean ± SEM, n = 5–8 per group.

To investigate how liraglutide, 7,8-DHF or liraglutide and 7,8-DHF co-treatment can modulate the volume of hippocampus and lateral ventricles (Fig. 6a), in vivo Magnetic Resonance Imaging (MRI) analyses were performed at the beginning of the experiment and after three months of treatment.

T2-weighted in vivo MRI analysis revealed that hippocampal volume increased in 7,8-DHF treated Wfs1 KO animals with age (p < 0.01) (Fig. 6b) and no significant differences were observed in other treatment groups. The volume of lateral ventricles increased in vehicle treated Wfs1 KO animals (p < 0.05) (Fig. 6c), indicating an enlargement of lateral ventricles in Wfs1 KO animals, which is a common pathology of neurodegeneration^16^. Treatment with liraglutide, 7,8-DHF or liraglutide and 7,8-DHF prevented lateral ventricles from enlargement (Fig. 6c).

Our next purpose was to assess inflammation in the hippocampus, therefore inflammatory marker *Ip10* levels were measured using gene expression analyses. *Ip10* mRNA levels were elevated in vehicle treated Wfs1 KO animals compared to vehicle treated WT animals (p < 0.05) (Fig. 6d) and treatment with liraglutide, 7,8-DHF or liraglutide and 7,8-DHF prevented the elevation of inflammation marker *Ip10* in the hippocampus. Additionally, a positive correlation was observed between *Ip10* mRNA levels and lateral ventricles volume increase (R^2^ = 0.580, p < 0.05) (Fig. 6e).

### Wfs1 KO rats show impaired gene expression in hippocampus

To investigate how liraglutide, 7,8-DHF or liraglutide and 7,8-DHF co-treatment can modulate the proliferation and cellular stress, we performed gene expression analyses of hippocampus, extracted from rats after 3 months of treatment, when the animals were 12.5 months old. The data were compared using factorial ANOVA test followed by Fisher’s LSD tests.

**Figure 7:**
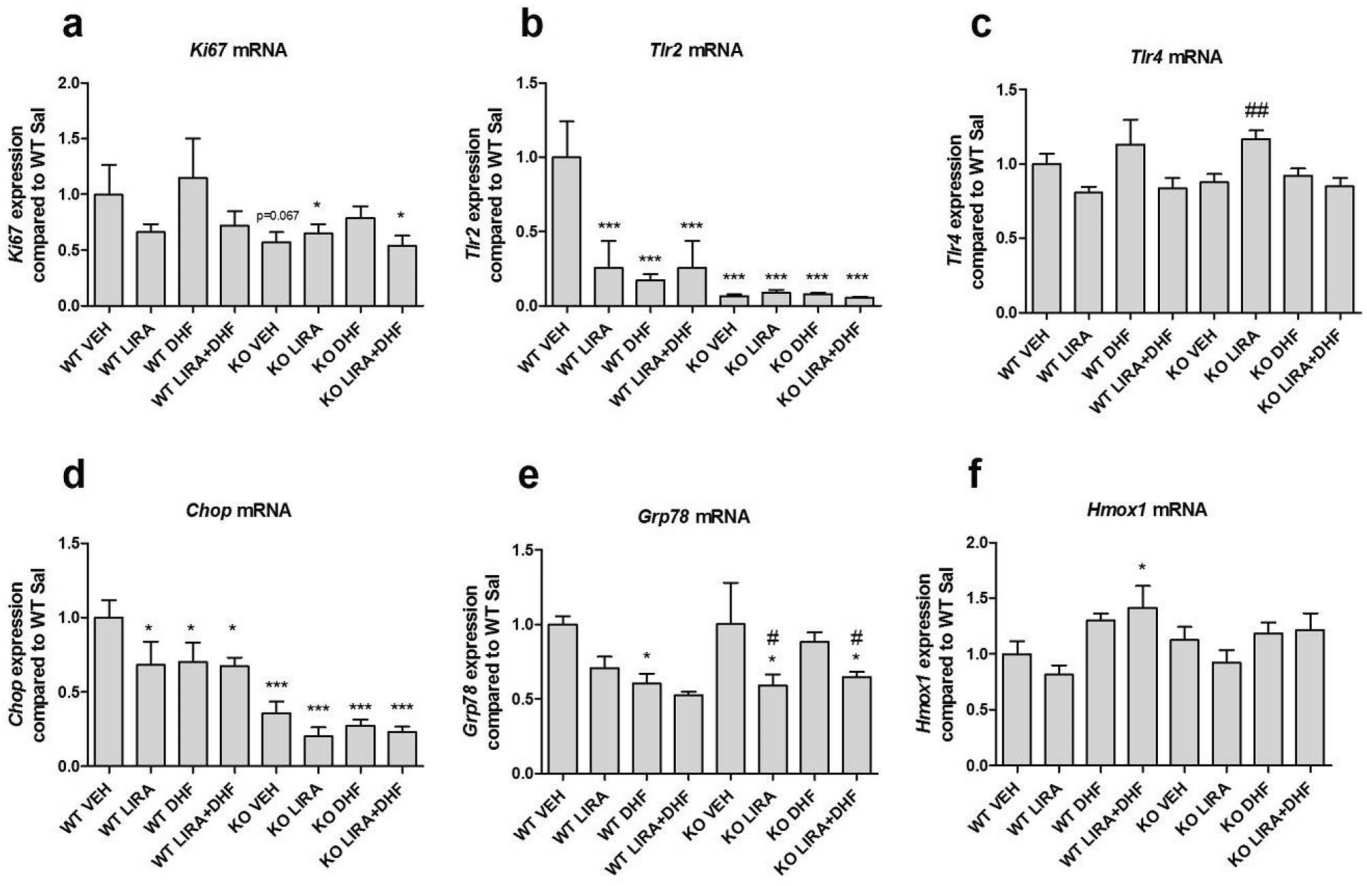
Hippocampus gene expression analyses from 12.5 months old animals after 3.5 months of treatment with liraglutide (LIRA), 7,8-DHF (DHF) and with their combination (LIRA + DHF). Gene expression analyses of hippocampus were performed to measure (**a**) *Ki67* mRNA, (**b**) *Tlr2* mRNA, (**c**) *Tlr4* mRNA (**d**) *Chop* mRNA, (**e**) *Grp78* mRNA, (**f**) *Hmox1*. (**a**) There was a tendency to proliferation marker *Ki67* downregulation in Wfs1 KO rats compared to WT VEH animals (p = 0.067) and treatment with 7,8-DHF protected Wfs1 KO animals’ hippocampus from proliferation downregulation. Gene expression data were compared using factorial ANOVA followed by Fisher’s LSD tests; *p < 0.05; **p < 0.01; ***p < 0.001 compared to WT vehicle animals and # p < 0.05; ## p < 0.01compared to KO vehicle animals. The data are presented as the mean ± SEM, n = 5–8 per group.

There was a tendency to downregulation of proliferation marker in Wfs1 KO rats (p = 0.067) (Fig. 7a) and treatment with 7,8-DHF protected Wfs1 KO animals’ hippocampus from proliferation downregulation. *Tlr2* deficiency causes lateral ventricles enlargement^17^, therefore our aim was to evaluate *Tlr2* levels in the hippocampus. *Tlr2* mRNA levels were downregulated in all treatment groups compared to WT vehicle treated animals (p < 0.001) (Fig. 7b) and treatments had no effect in Wfs1 KO animals. In contrast to *Tlr2* levels, *Tlr4* mRNA levels stayed unchanged between genotypes; although liraglutide treatment increased *Tlr4* mRNA levels in Wfs1 KO animals (p < 0.01) (Fig. 7c). Next, we found that in Wfs1 KO animals *Chop* mRNA levels are downregulated compared to WT littermates and surprisingly liraglutide, 7,8-DHF and their co-treatment decreased *Chop* mRNA expression in WT animals’ hippocampus (Fig. 7d).

*Grp78* is the key mediator of the UPR pathway and it was downregulated in WT animals treated with 7,8-DHF (p < 0.01) (Fig. 7e). *Grp78* was also downregulated in Wfs1 KO animals treated with liraglutide or with liraglutide and 7,8-DHF combination (p < 0.05) (Fig. 7e).

Expression of heme-oxygenase-1, *Hmox1* which is an important marker of oxidative stress was elevated in WT animals treated with 7,8-DHF and liraglutide combination (p < 0.05) (Fig. 7f).

## Discussion

The aim of this study was to investigate if BDNF mimetic 7,8-DHF alone or in the combination with GLP-1 receptor agonist liraglutide has a neuroprotective effect or even can induce regeneration in WS rat model. Therefore, we took 9 months old Wfs1 KO rats and their WT controls and treated them daily with vehicle solution, liraglutide, 7,8-DHF or with the combination of both liraglutide and 7,8-DHF up to the age of 12.5 months.

Liraglutide or liraglutide and 7,8-DHF co-treatment slightly reduced body weight in both genotypes during the first week of the treatment (Fig. 1a,b). 7,8-DHF treatment alone had no effect on body weight, although it has been shown that in vitro 7,8-DHF reduces fat production and fat buildup^18^. However, expected anti-obesity effect of 7,8-DHF was not observed in the current study. Additionally, a 3.5-month treatment with liraglutide or its co-treatment with 7,8-DHF prevented the progression of hyperglycemia in WS rats (Fig. 1c). Liraglutide antidiabetic effect is in agreement with our previous studies^4,5^. 7,8-DHF treatment alone had no effect on the diabetic phenotype, even though previous research has reported 7,8-DHF ability to lower fasting blood glucose^19^. Based on these results, 7,8-DHF is safe to use and does not affect the antidiabetic effect of liraglutide. Thus, co-treatment with 7,8-DHF and liraglutide may have greater neuroprotective effect than liraglutide alone, without affecting liraglutide beneficial effect on diabetic phenotype. This is important, as our main aim was to investigate the neuroprotective effect of 7,8-DHF alone or in combination with liraglutide.

Recently longitudinal follow up MR imaging has showed optic nerve atrophy development in time in a cohort of WS patients^20^ and additionally in WS post mortem study optic nerve degeneration in the inferior and temporal quadrants has been reported^21^. Similarly, we have described degenerative processes in 15-month-old WS rats’ optic nerves and resultant atrophy, although it is not known when the degenerative processes start, therefore we monitored in vivo changes in WS rats’ visual acuity over time. Visual acuity was already lower in 9 months old WS rats as compared to WT rats of the same age (Fig. 2a) and at the age of 11.5 months, WS rats’ visual acuity was even more deteriorated, which indicates clear degenerative processes in the visual pathway (Fig. 2b). Treatment with liraglutide, 7,8-DHF or with their combination kept WS rats’ visual acuity at the same level with WT control animals (Fig. 2b). Surprisingly, co-treatment synergistic effect was detected in improved visual acuity in WS rats (Fig. 2d). Liraglutide protective effect to visual pathway is in accordance with our previous results^5^ and 7,8-DHF has also shown to be sufficient to rescue visual function in a model of inherited blindness^22^.

Next, to reveal ultrastructural changes in optic nerve fibers ex vivo, transmission electron microscopy (TEM) was performed. We have shown degenerative processes in 15-month-old Wfs1 KO rats’ optic nerves^3^ and the following protective effect of liraglutide treatment^5^. Correspondingly, here we found decreased axon area per field of view (FOV) (Fig. 3j) in Wfs1 KO animals, which indicates optic nerve atrophy. Additionally, we observed a novel finding, axonal g-ratio was decreased in Wfs1 KO animals compared to WT control animals (Fig. 3l). The decrease in the value for g-ratio in Wfs1 KO animals could be explained by increased myelin area, which indicates degenerative processes in myelin, where myelin lamina decompacts (Fig. 3l). Based on well-established pathological characteristics and grading system of degenerating nerve fibers^1^ we describe increased ratio of degenerating fibers in vehicle treated Wfs1 KO rats optic nerves (Fig. 4g). Treatment of Wfs1 KO rats with liraglutide, 7,8-DHF, and co-treatment with liraglutide and 7,8-DHF halts optic nerve fiber degeneration which displays clear evidence for neuroprotection (Fig. 4g). These observations are in accordance with our previous study^2^. Similar effect of GLP1 receptor agonist is observed in the optic nerve crush model^3^. Additionally we performed morphometric analysis of remyelinating fibers which have the characteristics to fibers observed during the developmental period: thinned myelin sheaths accompanied by large inner tongue process^1,4^. Between the different treatments, the ratio of remyelinating fibers remained stable for WT animal optic nerves (Fig. 4p). In the Wfs1 KO animals, we observe severe decrease in remyelinating fibers, which could contribute to accelerated degeneration of the optic nerve. Based on morphometric analysis of the optic nerves treated with liraglutide and liraglutide and 7,8 DHF our results suggest induced remyelination in Wfs1 KO rats’ optic nerve fibers. Taken together, the increased prevalence of smaller axons (Fig. 3i,j) with degenerating optic nerve fiber, and disruption of myelin sheath integrity (Fig. 4a–g) are likely to be the cause of gradual loss of visual acuity as seen in 11.5 months old Wfs1 KO animals (Fig. 2b,d). Similarly, disruptions and abnormalities in axon caliber, myelin sheath integrity, and in visual function have been seen after partial injury to optic nerve^23^, which suggests a similar course of neurodegeneration in both cases. Treatment with liraglutide, 7,8-DHF and their combination were clearly protecting Wfs1 KO rats’ optic nerves and thus the animals maintained visual acuity similar to WT control animals.

All treatments have relatively similar neuroprotective effect on visual pathway, additionally co-treatment’s synergistic effect was detected in improved visual acuity (Fig. 2d), increased average axon area per FOV (Fig. 3j) and in the increased ratio of remyelinating axons (Fig. 4p). It seems that co-treatment is slightly more effective on visual pathway than the treatments solely. Although such synergistic co-treatment effect of liraglutide and 7,8-DHF has not been reported before and therefore these data must be interpreted with caution as further research is required to study this regenerating effect more precisely.

WS is also characterized by various neurological symptoms, such as cognitive decline, memory impairment, and learning difficulties^24,25^. As aging and neurodegeneration are mostly associated with a decrease in spatial memory and cognition, the purpose of the current study was to evaluate WS rats’ spatial learning abilities in Morris water maze experiment. WS rats needed more time to reach the platform on the probe day than WT controls, which indicates WS rats’ cognitive decline (Fig. 5j). Treatment with liraglutide, 7,8-DHF or their combination improved performance during the probe day (Fig. 5j). Thus, liraglutide and DHF improved the memory task. The present findings are consistent with other research which found that treatment with liraglutide^26,27^ or with 7,8-DHF^13^ was able to improve learning and memory in different rat models.

Reduced cognitive function is strongly correlated with hippocampal shrinkage^16^. Thus, we hypothesized that a decrease in the volume of hippocampus would be present in WS rats as well. Surprisingly, after three-month treatment the volume of hippocampus increased in 7,8-DHF treated WS rats and no significant differences were observed in other treatment groups (Fig. 6b). The increase in the volume of hippocampus could be related to increased neuronal proliferation as seen in increased *Ki67* mRNA expression in 7,8-DHF treated WS rats (Fig. 7a). Additionally, there was a tendency to increased proliferation in 7,8-DHF treated WT controls. The disagreement between our results and the hypothesis that a decrease in the volume of hippocampus would be present in WS rats could be due to the young age of animals. Decreased volume of the hippocampus was shown in 14 months old rats^28^ while just 12 months old rats were used here, therefore atrophy of the hippocampus was not detected and possibly the shrinkage of the hippocampus begins later in life. The results on hippocampal volume in WS patients are rather controversial as a decrease in hippocampal volume was found in one study^29^, while no changes were observed in other studies^30–32^, therefore further research should be done to clarify this issue.

Next, we monitored changes in animals’ lateral ventricles volume over time. Quantitative in vivo MRI analysis revealed ventricular enlargement in WS rats (Fig. 6c). Ventricular enlargement results from brain parenchymal shrinkage^33,34^ and it is accompanied by aging^28^ and neurodegeneration^35–37^. Additionally ventricular volume increase is in correlation with cognitive decline^38^. Indeed, here we also found that WS rats have cognitive decline and their lateral ventricles volume has increased. Ventricular volume increase could indicate neuronal loss in tissues surrounding brain ventricles. Therefore, further research is required to study brain ventricles and the surrounding brain regions in more detail.

Since inflammation is accompanied with neurodegeneration, our next purpose was to assess the inflammatory response in the hippocampus. Inflammatory cytokine *Ip10* mRNA expression was elevated in WS rats hippocampus (Fig. 6d), which indicates increased inflammation and is in agreement with our previous studies showing elevated inflammation in WS rats’ pancreatic beta cells^3^ and in the brainstem^5^. Additionally, there was a positive correlation between lateral ventricles volume increase and *Ip10* mRNA expression (Fig. 6e). Increased expression of *Ip10* is also reported in Alzheimer’s disease^39^. Treatment with liraglutide, 7,8-DHF or with their combination reduced *Ip10* in Wfs1 KO animals’ hippocampus. Thus, suggesting that cellular inflammation is reduced in the hippocampus after treatment with liraglutide, 7,8-DHF or with their combination.

As our results showed that WS rats have cognitive impairment and ventricle enlargement, our aim was to evaluate *Tlr2* levels in the hippocampus. *Tlr2* mRNA levels were downregulated in WS rats (Fig. 7b) which support the association between Tlr2 deficiency and lateral ventricles volume increase. Similar increase in lateral ventricles volume and cognitive impairment was also observed in Tlr2 KO animals^17^. In contrast to *Tlr2* levels, *Tlr4* mRNA levels stayed unchanged between genotypes. Tlr2 deficiency in mice is associated with impaired hippocampal neurogenesis, whereas the absence of Tlr4 resulted in enhanced proliferation and neuronal differentiation^40^. Thus, our results confirm that Tlr2 and Tlr4 might have distinct and opposing functions. Furthermore, we found that *Chop* mRNA levels were downregulated in WS rats compared to WT controls (Fig. 7d). Chop deficiency is associated with increased cell apoptosis and impaired memory performance, it can thus be suggested that Chop has a protective role in the hippocampus^41^. Interestingly *Grp78* expression was opposite between genotypes (Fig. 7e). In WT animals 7,8-DHF treatment decreased *Grp78* expression while in WS rats, 7,8-DHF had no effect on *Grp78* mRNA expression. *Hmox1* is an important marker of oxidative stress and was elevated in WT animals treated with the combination of 7,8-DHF and liraglutide (Fig. 7f). Moreover, there was a tendency to increased *Hmox1* levels in 7,8-DHF treatment groups in both genotypes and liraglutide treatment was not reversing 7,8-DHF effect. 7,8-DHF antioxidant properties could be possibly induced via upregulation of Nrf2-dependent *Hmox1* expression^42^.

In summary we show here that treatment with liraglutide inhibited the development of diabetes in WS rats as seen in previous studies^4,5^. 7,8-DHF treatment alone had no effect on the diabetic phenotype, and importantly in co-treatment, 7,8-DHF was not affecting liraglutide beneficial effect on diabetic phenotype. Based on these results, 7,8-DHF and liraglutide co-treatment is safe and therefore neuroprotective effect in the visual pathway and in the hippocampus was investigated. WS rats had decline in visual acuity that lead to degenerative processes in 12.5 months old WS rats’ optic nerves. By the age of 12 months WS rats had also developed cognitive decline, ventricular enlargement and inflammation, these symptoms are also the symptoms of AD^39,43–46^, which suggests a similar course of neurodegeneration in both diseases. Treatment with liraglutide, 7,8-DHF co-treatment with liraglutide and 7,8-DHF was effective in preventing these neurodegeneration symptoms in WS rat. Importantly 7,8-DHF did not reverse liraglutide effect or vice versa and additionally 7,8-DHF and liraglutide co-treatment had synergistic effect as seen in improved visual acuity, increased axon area and increased number of remyelinating axons in WS rats. These results show that treatment with liraglutide, 7,8-DHF or co-treatment with liraglutide and 7,8-DHF had a neuroprotective effect in WS rats and thus may have a similar effect in WS patients.

## Materials and methods

### Animals

All experimental protocols were approved by the Estonian Project Authorisation Committee for Animal Experiments (No 155, 6th of January 2020), and all experiments were performed in accordance with the European Communities Directive of September 2010 (2010/63/EU) and the study was carried out in compliance with the ARRIVE guidelines.

Male Wfs1 KO and WT littermate control rats were used in this study. Animals’ breeding, genotyping, and housing conditions are described in our previous study^3^. The rats were 9 months old at the beginning of the experiment. Rats were randomly allocated into eight experimental groups: (WT VEH, n = 5; WT LIRA, n = 5; WT DHF, n = 5; WT LIRA + DHF, n = 6; KO VEH, n = 6; KO LIRA, n = 7; KO DHF, n = 7; KO LIRA + DHF, n = 8). Vehicle treated animals served as control animals. Different baseline between experimental groups in fasting blood glucose and in visual acuity can be explained with the fact that that rats were randomly allocated into eight experimental groups before the beginning of the treatment. There is usually increased variability in in vivo animal experiments; therefore, we used in fasting blood glucose, visual acuity and MRI volumetric measurements repeated measures and compared only the same animals’ data before and after.

Drugs were administered once a day between 8 and 11 a.m. Rats were weighed once a week, and their base blood sugar level was measured once a month from the tail vein using a handheld glucometer (Accu-Check Go, Roche, Germany). Liraglutide (LIRA) from commercially available pens (NovaNordisk) was diluted with 0,9% saline and injected s.c. at a dose 0.4 mg/kg, vehicle (0.9% saline) was administered in a volume 1 ml/kg. BDNF mimetic 7,8-dihydroxyflavone (7,8-DHF, catalog # D1916, Tokyo Chemical Industry CO Ltd, Tokyo, Japan) was first dissolved in DMSO (at concentration 400 mg/ml), and this DMSO solution was further diluted 1:20 with PEG-300/PBS mix (1:1). Final composition of 7,8-DHF injection solution was 20 mg/ml 7,8-DHF in 5% DMSO, 47.5% PEG-300, 47.5%PBS. The drug or the corresponding vehicle (VEH) was administered subcutaneously in a volume of 0.25 ml/kg, dose of 7,8-DHF was 5 mg/kg.

To avoid hyperglycemia-induced symptoms, supportive insulin treatment (100 IU/ml, Levemir, Novo Nordisk, Denmark) was initiated in hyperglycemic Wfs1 KO rats. Animals with a blood glucose level of 10 mmol/L or more received 2 IU/kg insulin and animals with a blood glucose level of 20 mmol/L or more received 6 IU/kg insulin twice per day injected subcutaneously.

Within 24 h after the last injection, the animals were sacrificed with an intraperitoneal injection of Euthasol vet (dose 300 mg/kg). Hippocampus was dissected, washed quickly with 0.9% saline and snap frozen in liquid nitrogen. Tissue samples were stored at -80 °C until analysis.

### Visual acuity estimation

To measure visual acuity, a virtual optomotor task (OptoMotry, Cerebral Mechanics Inc) was used. The animal was placed inside the test chamber platform surrounded by four 19″ computer monitors displaying vertical sinusoidal gratings with the animal's viewpoint at the center of a ‘virtual cylinder’. Animal head was maintained at the center of the virtual cylinder and the grating was rotated 12°/s to induce head movements in the same direction as the visual motion. The operator selects YES or NO depending on if there was head-tracking behavior or not to follow the moving gratings. If operator was selecting YES, the grating frequency was increased, but rotation speed remained the same. Using a OptoMotry HD 2.1 software and manufacturer validated single frequency threshold staircase protocol, head-tracking behavior was protocolled until there were no movements, which indicates the maximum visual acuity threshold (in cycles per degree; c/d) of the animal as the highest grating frequency on which the animal was responding. Clockwise rotation was detected by the left eye and anti-clockwise rotation by the right eye. Visual acuity data are presented as the mean of clockwise and anti-clockwise testing^47,48^.

### Transmission electron microscopy

The optic nerves from each animal were immersion-fixed in 2.5% glutaraldehyde (AppliChem)/4% paraformaldehyde (Merck)/90 mM Na-cacodylate solution (AppliChem) and kept at 4 °C until further washed with 90 mM Na-cacodylate buffer (pH 7.4). Nerves were subsequently fixed in a reduced 1% OsO_4_ (AppliChem) solution containing 1% potassium ferrocyanide (Roch)/90 mM Na-cacodylate for 2 days at 4 °C. Subsequently, nerves were washed with 90 mM Na-cacodylate, dehydrated in graded ethanol series and embedded in epoxy resin (medium hardness, TAAB) according to manufacturer's protocol. Preparation of epoxy embedded nerves for electron microscopy were performed as described previously^3^. Electron microscopic analysis of specimens was performed using a Tecnai G^2^ Spirit TWIN/BioTWIN transmission electron microscope (FEI, Netherlands).

### Analysis and morphometric evaluation of optic nerve fibers

Images for morphometric evaluation and nerve fiber measurements were captured with pixel size corresponding to 0. (4) × 0. (4) nm giving the field of view of 220.472 μm^2^. Cross sections of nerves were divided into 10–11 non-overlapping random regions to minimize the variation in fiber size distributions. On average 1200 nerve fibers were counted from individual rats (4–7 animals from each group) whereas only fibers whose contour was completely within field of view were counted. All nerve fiber and axon measurements were performed using Fiji software^49^. Axon area measurements were performed on binned (binning factor 10 × 10) and gaussian blurred (sigma radius 3) images using Fiji plugin AxonJ^50^. Average axon area per field of view was calculated as sum of individual axon areas per field of view. Average myelin areas were measured as the area of black pixels in binary converted images per field of view. Area based g-ratio (axon area divided by axon area plus myelin area) were used.

Nerve fibers were considered degenerating if one or more following pathological manifestations were present: severe outfoldings and infoldings of the myelin sheath leading to constriction of axoplasm; axonal vacuolization; severe hypermyelination leading to axonal compression/atrophy; abnormally electron dense/lucent axoplasm; myelin lamellae breakdown accompanied by axonal atrophy. Following parameters were taken and supplemented from^15^. Ratios of degenerating fibers were calculated as the ratio of degenerating fibers to all counted fibers per field of view.

In order to quantify remyelinating axons the fibers must display the following distinctive features few myelin lamellae and the large inner tongue process containing decompacted myelin lamellae as described^15^. Remyelinating fibers were calculated as the ratio to all counted fibers per field of view.

### Morris water maze

The rats were allowed to adapt to experimental room for two hours on the experiment days^46^. The water maze consisted of 180 cm diameter plastic pool filled with water at room temperature (20–22 °C). The escape platform (diameter, 14 cm; height, 29 cm) was placed on a fixed position in the center of one quadrant, 35 cm from the perimeter, and was hidden 1 cm beneath the water surface. Six black and white cues were fixed around water maze. Water was painted black using tempera paint to allow automatic video recording of albino rats using EthoVision software (Noldus Information Technology, Wageningen, the Netherlands). Acquisition phase lasted four days and four trials per day were performed. On each trial, animal was placed into the pool at pseudo-random location facing the wall of the pool. Rats were allowed to find a platform during 60 s session and were guided to the platform if failed to find the platform. Rats had to remain on the platform for 30 s before they were rescued and placed on the cage filled with paper towels. Next session started after 60 s rest period. Latency to find the platform, time in target quadrant and swimming speed was recorded during acquisition phase. The platform was removed on the probe day (day 5 of the experiment), rats were released at unfamiliar location (same for all animals) and allowed to search for platform for 60 s. Time to find the platform, percentage of time in target quadrant and swimming speed was recorded.

### In vivo magnetic resonance imaging

9- and 12-months old rats in vivo magnetic resonance imaging was performed as described previously by our group^3^. The volume of hippocampus was segmented manually using ITK-SNAP (V3.6.0)^51^. The Scalable Brain Atlas^52^ was used to determine the segmentation start, for the hippocampus, segmentation began from bregma − 1.45 mm and continued to − 5.82 mm until reaching the most caudal end of the thalamus. In all animals, the hippocampus was measured on consecutive 10 slices. From the same images, lateral ventricles were delineated based on the nearly saturated bright signal in T2-weighted images from bregma – 3.32 mm to – 4.57 mm.

### RNA isolation, cDNA synthesis and gene expression analyses

The hippocampus was homogenized using Precellys lysing Kit CK14 (Bertin Instruments) and Precellys homogenizer (Bertin Instruments). RNA from hippocampus was isolated using Direct-zol RNA MiniPrep (Zymo Research), according to the manufacturer’s protocol. SuperScrip III Reverse Transcriptase (Invitrogen, USA) and random hexamers were used to synthesize cDNA according to the manufacturer’s protocol. Taqman Gene Expression Mastermix (Thermo Fisher Scientific) and following TaqMan Gene Expression Assays were used for real-time quantitative PCR: *Ip10* (Rn01413889_g1), *Ki67* (Rn01451446_m1), *Tlr2* (Rn02133647_s1), *Tlr4* (Rn00569848_m1), *Chop* (Rn00492098_g1), *Grp78* (Rn00565250_m1) and *Hmox1* (Rn00561387_m1). *Hprt1* (Rn01527840_m1) was used as an internal control and 2^−∆Ct^ method was used for relative quantification.

### Data analysis

GraphPad Prism version 5 software (GraphPad Software Inc., San Diego, CA, USA) and STATISTICA 8 package (StatSoft Inc, Tulsa, OK, USA) was used for statistical analysis. The data were compared using repeated measures ANOVA followed by Bonferroni post hoc tests or factorial ANOVA followed by Fisher’s LSD tests. Data are presented as means and standard error of the mean (SEM), p value less than 0.05 was considered statistically significant.
